# Pyrosequencing detects human and animal pathogenic taxa in the grapevine endosphere

**DOI:** 10.3389/fmicb.2014.00327

**Published:** 2014-07-08

**Authors:** Sohail Yousaf, Daniela Bulgari, Alessandro Bergna, Michael Pancher, Fabio Quaglino, Paola Casati, Andrea Campisano

**Affiliations:** ^1^Sustainable Agro-Ecosystems and Bioresources Department, Research and Innovation Centre, Fondazione Edmund MachSan Michele all'Adige, Italy; ^2^Department of Environmental Sciences, Faculty of Biological Sciences, Quaid-i-Azam UniversityIslamabad, Pakistan; ^3^Dipartimento di Scienze Agrarie e Ambientali - Produzione, Territorio, Agroenergia, Università degli Studi di MilanoMilano, Italy

**Keywords:** endosphere, grapevine, pathogens, bacteria, pyrosequencing

## Abstract

Generally, plants are not considered as hosts for human and animal pathogens (HAP). The recent produce-associated outbreaks of food-borne diseases have drawn attention toward significant deficiencies in our understanding of the ecology of HAP, and their potential for interkingdom transfer. To examine the association of microorganisms classified as HAP with plants, we surveyed the presence and distribution of HAP bacterial taxa (henceforth HAPT, for brevity's sake) in the endosphere of grapevine (*Vitis vinifera* L.) both in the plant stems and leaves. An enrichment protocol was used on leaves to detect taxa with very low abundance in undisturbed tissues. We used pyrosequencing and phylogenetic analyses of the 16S rDNA gene. We identified several HAPT, and focused on four genera (*Propionibacterium, Staphylococcus, Clostridium*, and *Burkholderia*). The majority of the bacterial sequences in the genus *Propionibacterium*, from grapevine leaf and stem, were identified as *P. acnes*. Clostridia were detected in leaves and stems, but their number was much higher in leaves after enrichment. HAPT were indentified both in leaves and wood of grapevines. This depicts the ability of these taxa to be internalized within plant tissues and maintain their population levels in a variety of environments. Our analysis highlighted the presence of HAPT in the grapevine endosphere and unexpected occurrence of these bacterial taxa in this atypical environment.

## Introduction

Endophytes (non-pathogenic microorganisms living inside plant tissues and cells) are common inhabitants of interior plant parts (the endosphere) universally present and found in all the species of plants studied up till now (Schulz and Boyle, 2006). Some endophytes are able to promote plant growth and to suppress plant diseases (Compant et al., 2005; Lugtenberg and Kamilova, 2009).

Intriguingly, among these endosphere-dwellers, we can occasionally find some human and animal pathogens (HAP) (Holden et al., 2009; Kirzinger et al., 2011). HAP not only contaminate plant surfaces, but also actively interact with plants and can colonize them as alternative hosts (Holden et al., 2009). Some human bacterial pathogens are capable to colonize inner plant tissues (Tyler and Triplett, 2008), a phenomenon that in most cases can be considered as an opportunistic exploitation of a short-term habitat (Campisano et al., 2014). Generally, pathogens are studied solely for their harmful impact on human and animal health, causing disease and epidemics. On the other hand, the regular interaction of human and animal carriers with their environment puts these pathogens in contact with alternative niches, including additional hosts (Enders et al., 1993; Lenz et al., 2003). It is then unsurprising that previous works found out well known and potential HAP undergoing an endophytitc stage in their lifestyle (Kirzinger et al., 2011). Scientific literature often reports that members of the family *Enterobactericeae*, including pathogenic *Salmonella* and *Shigella* genus strains, *Vibrio cholerae* strains, and the human opportunistic pathogen *Pseudomonas aeruginosa* were found on plants or inside plants (Akhtyamova, 2013). *Salmonella enterica* strains have been isolated as endophytic colonizers of barley roots, spreading to the rhizodermis layers (Kutter et al., 2006).

Enteric bacterial pathogens, usually transmitted through foods, are well adapted to vertebrate hosts and generally colonize the gut (Wagenaar, 2008). Some have humans as their principal host, while many others are persistent in animal populations, adapted to a particular reservoir or environment, and affect humans only incidentally (Lynch et al., 2009). HAP on plants are generally thought of as having a reservoir in the intestines of a vertebrate host and, once discarded in manure, coming into direct or indirect contact with epigeous or hypogeous plant tissues in a variety of ways. Traditionally, they were considered to be fleeting on plant surfaces, persisting inertly in cracks, wounds, and stomatal openings. They were considered unable to aggressively modify or to communicate with the plant. However, it is now apparent that enteric bacteria do not just land on and reside in plants. These pathogens can stick tightly to produce, multiply, and enter into the tissues of leaves or fruits, in some cases even moving into inner plant parts (Berger et al., 2010; Erickson, 2012). Whether endophytic growth may actually be part of HAP life cycle is under debate. Findings such as those mentioned before, reporting an endophytic stage for HAP, would explain why existing surface decontamination procedures may be inefficient in removing contaminants from plant produce (Rosenblueth and Martinez-Romero, 2006; Teplitski et al., 2009; Saldaña et al., 2011; Barak and Schroeder, 2012; Olaimat and Holley, 2012). The diffusion of HAP in ecosystems are of scrupulous importance from the perspectives of both biology and evolution of cross-kingdom pathogenesis and/or adaptation (Lenz et al., 2003; Kirzinger et al., 2011). While the majority of published research has focused on describing the enteric HAP, there is no doubt that other HAP can also interact with plants as part of their life-cycle. Here, we surveyed the presence and distribution of HAP taxa (HAPT) in the endosphere of grapevine (*Vitis vinifera* L.). We used pyrosequencing of the bacterial 16S rDNA gene to identify sequences belonging to genera where HAP are abundant. We present the first (to the authors' best knowledge) report of the occurrence of bacteria in taxa potentially pathogenic to human and animals in the grapevine endosphere.

## Materials and methods

### Sample collection and DNA extraction

Grapevines samples were taken in Northern Italy. For stem endophytes analysis, 12 plants (7 plants cv Chardonnay and 5 plants cv Merlot) were sampled from vineyards in Trentino, Italy with farmers permission (sites are mapped here: http://goo.gl/maps/7AI7j) during the fall of 2010, from October 27th to November 11th. One lateral shoot per plant was removed and stored briefly at 5–10°C. Plant material was pre-processed as described previously (Pancher et al., 2012) and DNA was extracted from surface-disinfected and aseptically peeled grapevine stems. Briefly, plant material was pulverized in sterile steel jars using liquid nitrogen and a mixer-mill. DNA was extracted from each sample using FastDNA spin kit for soil and a FastPrep-24 mixer (MP Biomedical, USA) according to standard manufacturer protocols. Plants used for leaf endophytes analysis (cv Barbera) were sampled from a vineyard in Lombardia, Italy (site is mapped here: http://goo.gl/5Nfh9R) on October 15th, 2007. Plant leaves were surface-sterilized as previously described (Bulgari et al., 2009) and aseptically prepared for DNA extraction. In a subset of leaf samples DNA isolation was preceded by a microbe enrichment strategy as described in previous studies (Jiao et al., 2006; Bulgari et al., 2009, 2011). Briefly, plant tissues were sterilized, grounded in liquid nitrogen, and aseptically incubated at 28°C for 12 h in gentle agitation in an enzymatic solution (0.1% macerozyme, 1% cellulase, 0.7M mannitol, 5 mM N-morpholinoethanesulfonic acid, 9 mM CaCl_2_, and 65 μM KH_2_PO_4_). In another subsample, DNA isolation was performed directly after surface sterilization. DNA was extracted for each sample according to the protocol described by Prince et al. (1993) modified by the addition of lysozyme (3 mg/ml), L-lysine (0.15 mol/l), EGTA (6 mmol/l, pH 8.0), and by the incubation at 37°C for 30 min, before the lysis step.

### Pyrosequencing of endophytic communities

To obtain amplicons for pyrosequencing, we amplified the 16S rDNA gene from each sample using High Fidelity FastStart DNA polymerase (Roche, USA) and the universal primers 799f/1520r with 454 adaptors and a sample-specific 10-mer barcode (designed following the instructions for Roche 454 technology^1^). These primers allow selective amplification of bacterial DNA, targeting 16S rDNA hypervariable regions v5-v9 (Chelius and Triplett, 2001) and minimize the chance of amplification of plastid DNA (Ghyselinck et al., 2013). The PCR product was separated on 1% agarose gel and gel-purified using Invitrogen PureLink (Invitrogen, USA). DNA was quantified via quantitative PCR using the Library quantification kit—Roche 454 titanium (KAPA Biosystems, USA) and pooled in a final amplicon library. The 454 pyrosequencing was carried out at the Sequencing Platform facility in Fondazione Edmund Mach, on the GS FLX+ system using the new XL+ chemistry dedicated to long reads of up to 800 bp, following the manufacturer's recommendations. The new XL+ chemistry coupled with the unidirectional sequencing strategy led to the sequencing of multiple variable regions on a single read and to overcome the bottleneck associated with a short read approach.

### Data analysis

**Figure 1:**
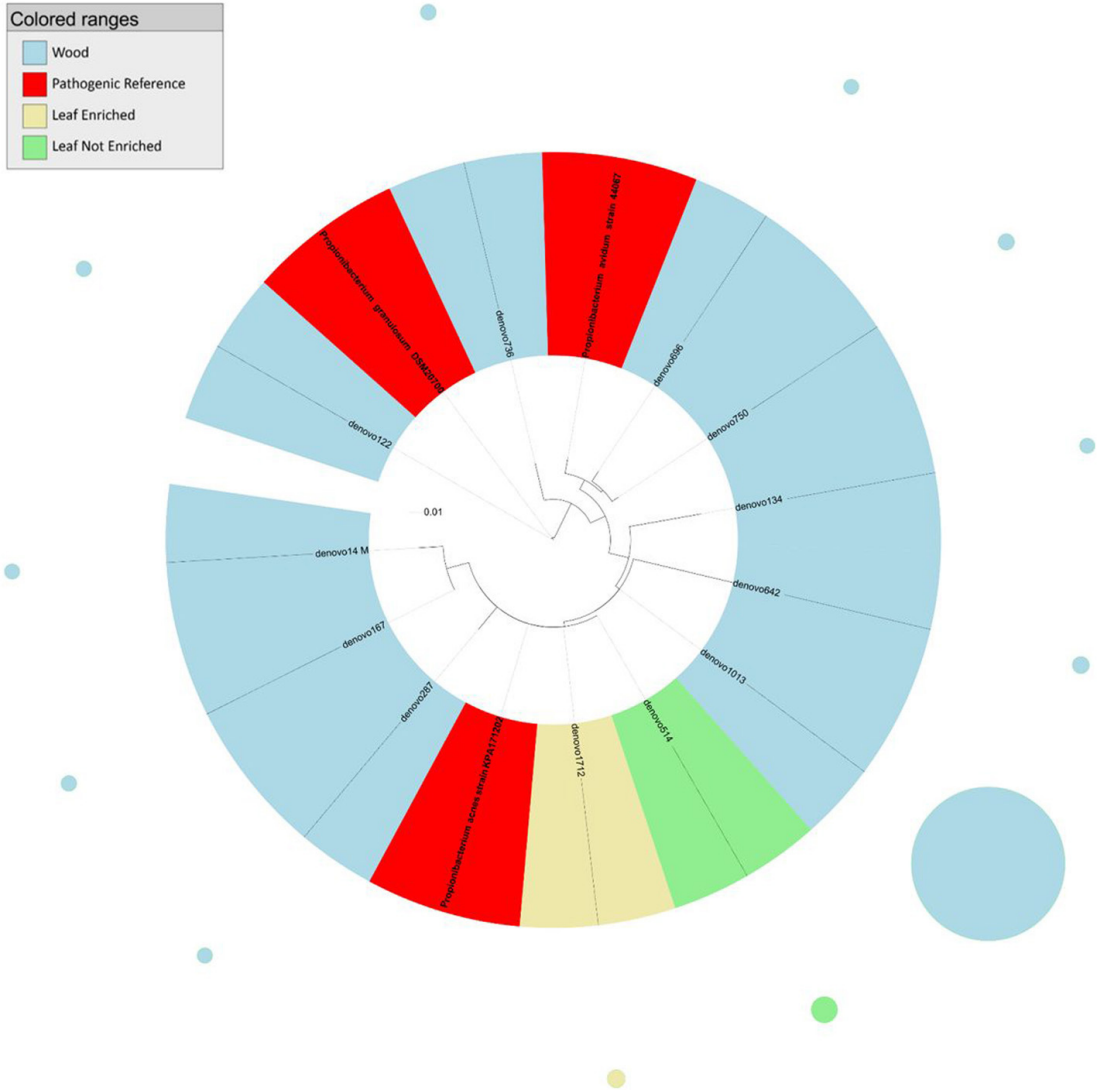
**Phylogenetic relationships based on partial 16S rDNA gene sequences obtained from pyrosequencing (this work) and closely related *Propionibacterium* sequences, retrieved from GenBank (Pathogenic and non-pathogenic Reference)**. The tree was built using a maximum likelihood method and rendered using iTOL. The relative abundance of each OTU is reported as circles and it is proportional to circle radius. Sequences tag and relative abundance circles were colored according to the source dataset.

**Figure 2:**
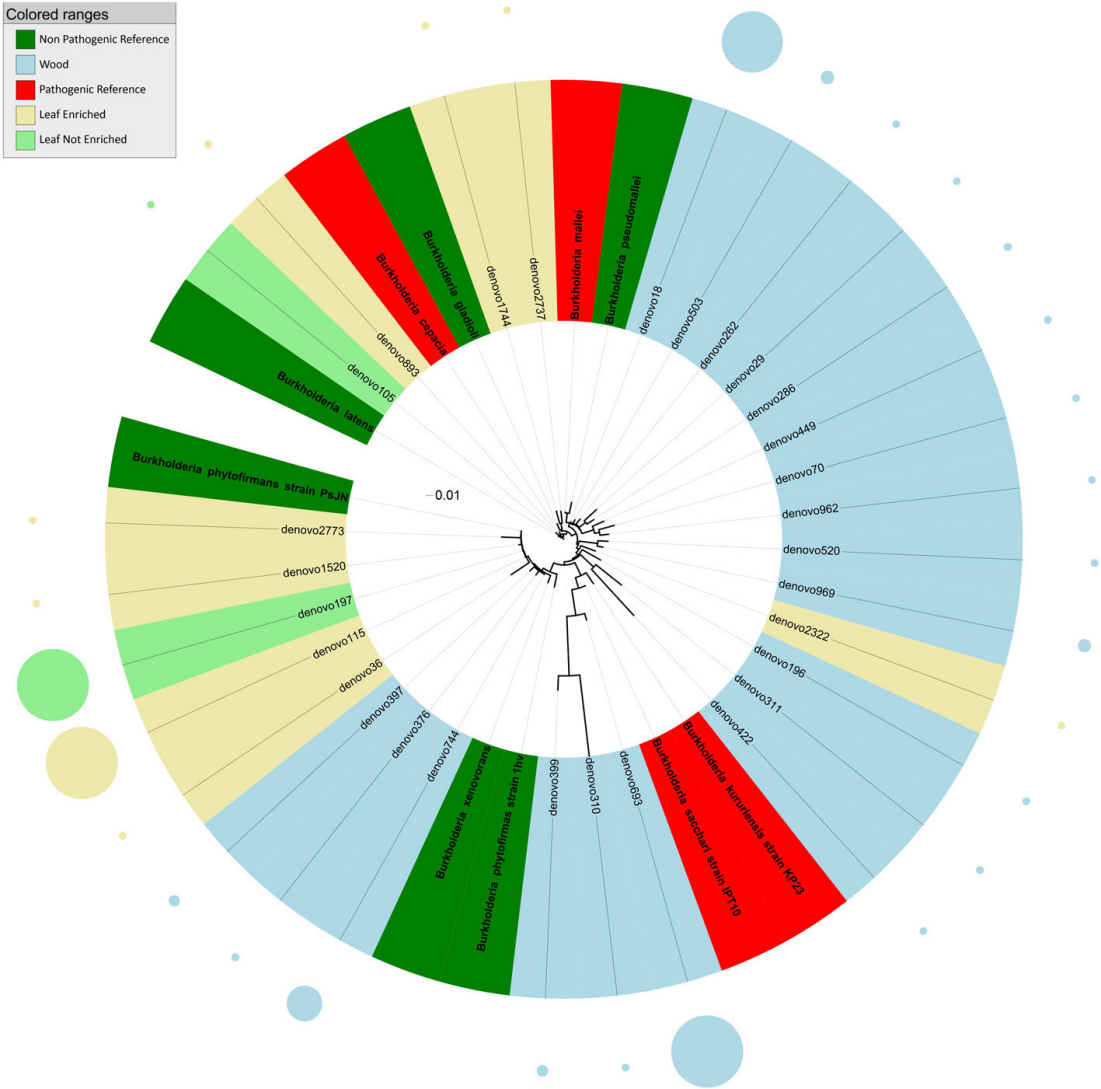
**Phylogenetic relationships based on partial 16S rDNA gene sequences obtained from pyrosequencing (this work) and closely related *Burkholderia* sequences, retrieved from GenBank (Pathogenic and non-pathogenic Reference)**. The tree was built using a maximum likelihood method and rendered using iTOL. The relative abundance of each OTU is reported as circles and it is proportional to circle radius. Sequences tag and relative abundance circles were colored according to the source dataset.

**Figure 3:**
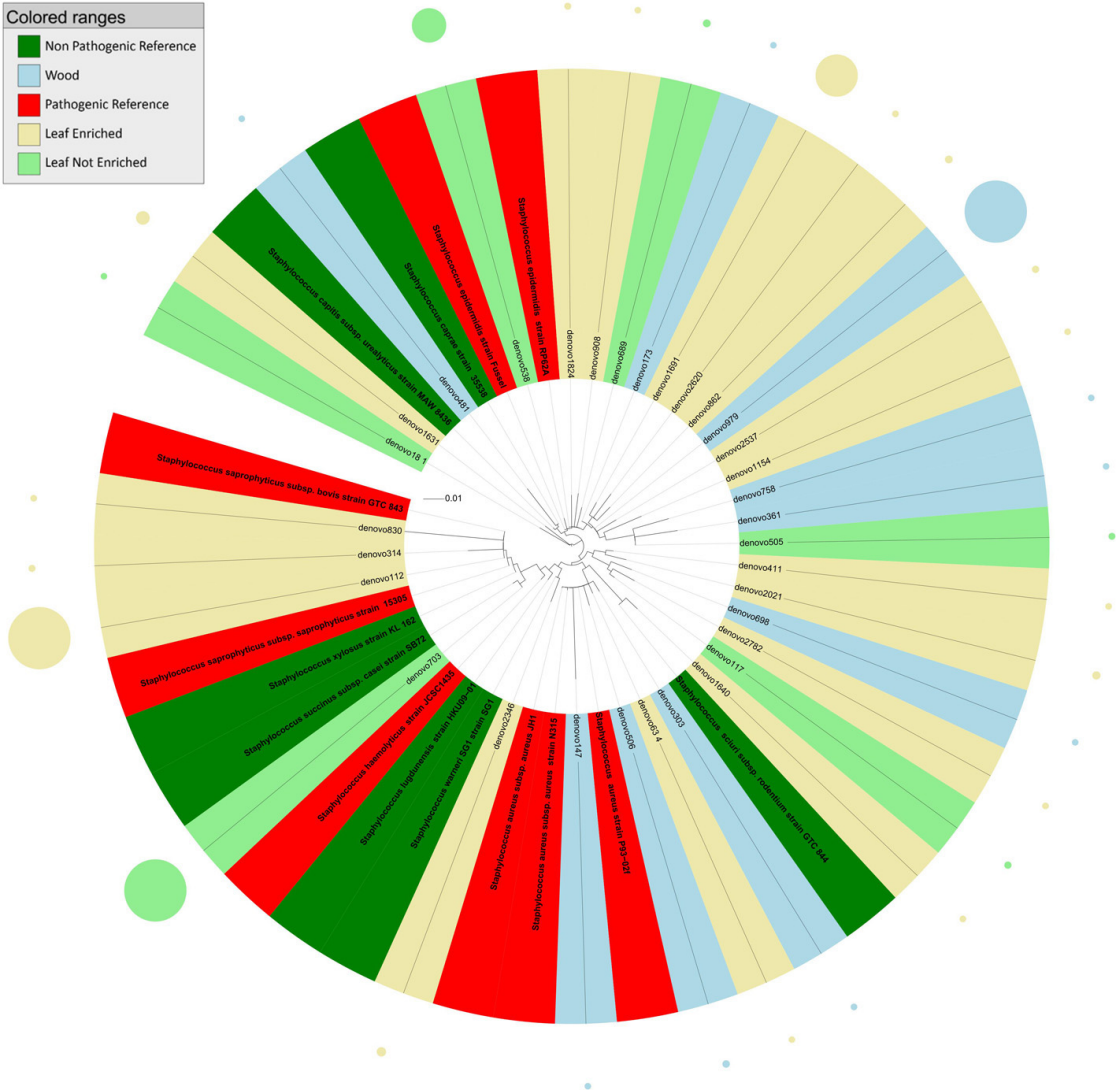
**Phylogenetic relationships based on partial 16S rDNA gene sequences obtained from pyrosequencing (this work) and closely related *Staphylococcus* sequences, retrieved from GenBank (Pathogenic and non-pathogenic Reference)**. The tree was built using a maximum likelihood method and rendered using iTOL. The relative abundance of each OTU is reported as circles and it is proportional to circle radius. Sequences tag and relative abundance circles were colored according to the source dataset.

**Figure 4:**
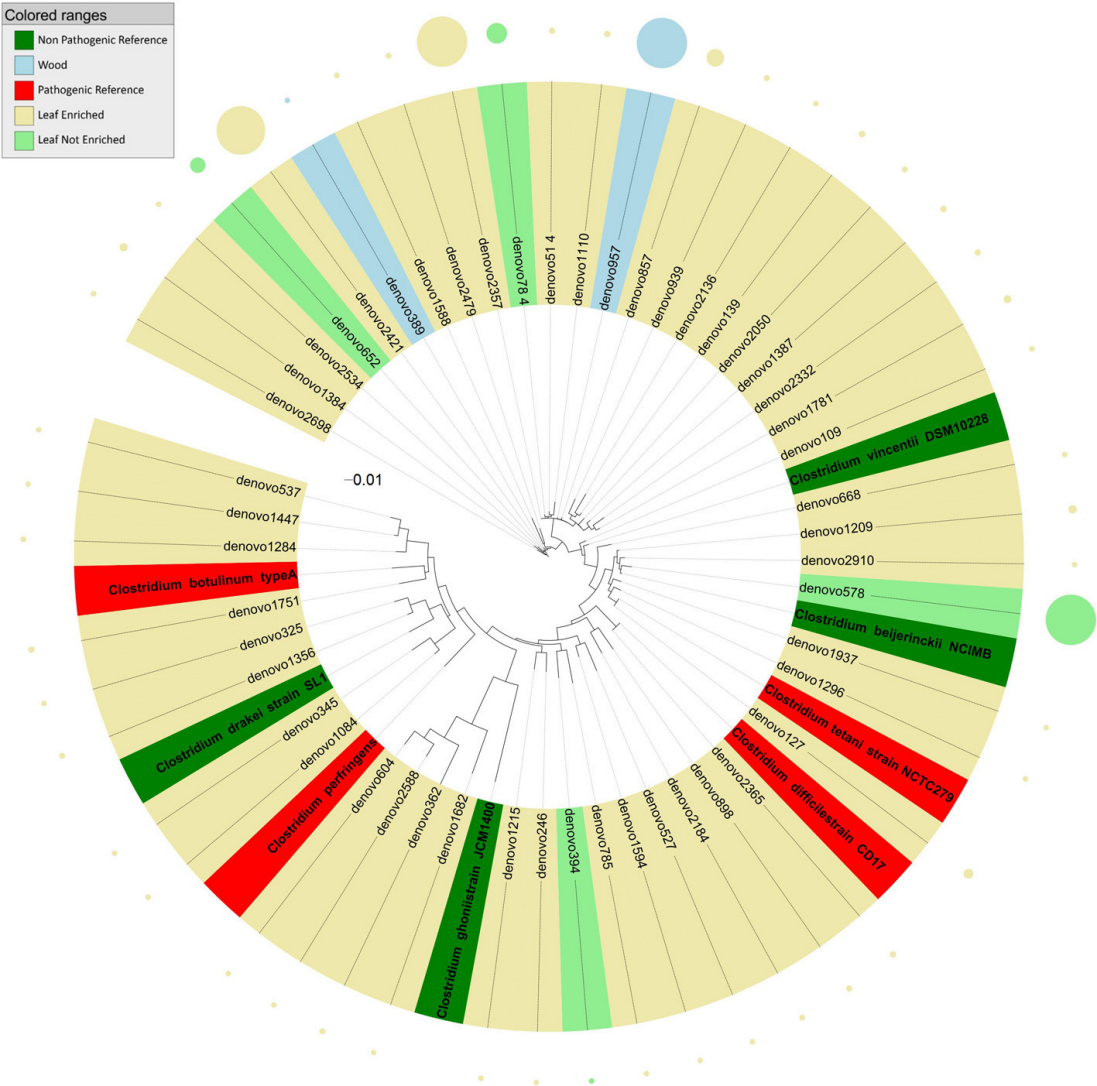
**Phylogenetic relationships based on partial 16S rDNA gene sequences obtained from pyrosequencing (this work) and closely related *Clostridium* sequences, retrieved from GenBank (Pathogenic and non-pathogenic Reference)**. The tree was built using a maximum likelihood method and rendered using iTOL. The relative abundance of each OTU is reported as circles and it is proportional to circle radius. Sequences tag and relative abundance circles were colored according to the source dataset.

We generated 16S rDNA gene sequences relative to endophytes from three batches of samples: leaf endophytes with bacterial enrichment, leaf endophytes without enrichment, and stem endophytes. We used Roche 454 GS FLX+ sequencing as described above and analyzed the sequencing output using a standard Qiime pipeline (Caporaso et al., 2010). As a first step we demultiplexed and filtered sequences on the basis of quality score and read length (only sequences with a minimum average score of 20 and length between 250 and 1000 bp were retained), in order to remove short and low quality reads. Moreover, in this step we removed sequences with more than 6 ambiguous bases or with homopolymers longer than 6 bases. We performed chimera identification and filtering using usearch61 (Edgar, 2010). After removing chimeras, we used uclust (Edgar, 2010) for clustering all the sequences into Operational Taxonomic Units (OTUs), by applying a similarity threshold of 97%. This is commonly used to represent species level similarity (although this threshold does not necessarily match with what is regarded as species for many microorganisms) (Crawford et al., 2009). For each OTU we picked a representative sequence using Qiime and we used these sequences to assign a taxonomical identity to each OTU using the RDP classifier (Wang et al., 2007). In this step we used a confidence threshold of 0.8 and *e*-value ≤ 0.001 against the Greengenes 97% reference data set. Based on taxonomical identity, we manually selected OTUs corresponding to HAPT from all three datasets. To understand the distance between these OTUs and database reference strains, we downloaded 16S rDNA sequences of HAP and non-pathogenic microorganisms from NCBI (www.ncbi.nlm.nih.gov) (Altschul et al., 1997) and aligned them to the representative sequences assigned to the same taxon in our datasets. For the alignment we used MUSCLE (Edgar, 2004; Caporaso et al., 2010) and filtered the alignment using Qiime. Then, we built the phylogenetic trees shown in Figures 1–**4** from these alignments using DNAML (Maximum Likelihood) in BioEdit (Hall, 1999) and rendered them using Itol (Letunic and Bork, 2006, 2011). Information on OTU type and abundance was co-displayed with the generated trees. OTUs were colored according to the origin of samples (types: stem, leaf enriched, leaf non-enriched, HAP reference strain, non-pathogenic reference strain). Abundance was displayed as circles for every OTU (abundance is proportional to the circle radius).

The 16S rDNA gene nucleotide sequences, representative of the OTUs identified in this study, are available with NCBI GenBank accession numbers KJ851800-KJ851922 (Supplementary Table 1).

## Results

We investigated the composition of bacterial endophytic communities in the grapevine endosphere (both in the leaf and the stem) by pyrosequencing the bacterial 16S rDNA gene. Sequence analysis revealed the presence of several OTUs (70 from enriched leaves, 13 from non-enriched leaves, and 40 from stems, see Table 1) classified as HAPT or non-pathogenic taxa closely related to HAPT. We focused our subsequent analysis on four genera containing potential pathogens: *Propionibacterium, Staphylococcus, Clostridium*, and *Burkholderia*.

**Table 1:** **Specifics for each datasets: number of samples collected, number of reads retained after quality filtering, OTUs retained after singleton removal, OTUs assigned to HAPTs and number of reads clustered in these OTUs**.

**Dataset**	**Plant samples**	**Total reads**	**Reads after filtering**	**total OTUs**	**OTUs of HAPT**				**Read of HAPT**			
					***Burkholderia***	***Clostridium***	***Propionibacterium***	***Staphylococcus***	***Burkholderia***	***Clostridium***	***Propionibacterium***	***Staphylococcus***
Leaf enriched	56	201517	175578	2937	8	44	1	17	3095	3394	92	2139
Leaf not enriched	42	214376	186114	724	2	4	1	6	248	41	150	1652
Wood	12	97399	84750	1069	19	2	10	9	2922	42	2371	2748

We inferred phylogenetic trees from the alignment of 16S rDNA nucleotide sequences. OTUs assigned to these taxa were identified, with varying abundance, in enriched and non-enriched leaves and in stems of grapevine. Most OTUs from grapevine leaves were assigned to genera *Clostridium* (44 and 4 OTUs from enriched- and not-enriched leaves, respectively) and *Staphylococcus* (17 and 6 OTUs from enriched- and not-enriched leaves, respectively), albeit the most abundant OTU from enriched leaf samples (denovo115) was associated with non-pathogenic species of the genus *Burkholderia* (Figure 2). The most abundant OTU from non-enriched leaves (denovo703) was assigned to a cluster including both pathogenic and non-pathogenic species of the genus *Staphylococcus*. On the other hand, most OTUs from endophytic bacteria in grapevine stems were assigned to genera *Burkholderia* (19 OTUs) and *Propionibacterium* (10 OTUs), although the most abundant OTU (denovo979) was taxonomically closer to pathogenic species *Staphylococcus epidermidis* (Figure 3).

The analysis of the phylogenetic tree of propionibacteria showed three main clades within the *Propionibacterium* genus (Figure 1). In detail, all sequences from grapevine leaves and most sequences from the stem clustered (cluster 1) with *Propionibacterium acnes* (which we previously demonstrated to be endocelluarly associated with grapevine stems, Campisano et al., 2014), while other sequences from the stem clustered with the species *P. granulosum* (cluster 2) and *P. avidum* (cluster 3).

Phylogenetic analysis indicated that sequences from grapevine endosphere assigned to the genus *Burkholderia* formed four distinct clusters (Figure 2). The first cluster included endophytic bacteria from grapevine leaves grouping with HAP and plant pathogens such as *B. latens*, *B. cepacia*, and *B. gladioli*. The second cluster included bacteria, identified exclusively in stems, grouping with *Burkholderia mallei* (HAP) and *B. pseudomallei* (non-pathogenic endophyte). The third cluster included endophytic bacteria from grapevine leaves and stem, grouping with environmental and endophytic bacteria such as *Burkholderia phytofirmans* and *B. xenovorans.* The forth cluster included HAP species of the genus *Burkholderia* (*B. kururiensis* and *B. sacchari*), and consisted of endophytic bacteria identified exclusively in grapevine wood tissues.

The tree obtained by analysis of 16S rDNA sequences assigned to the genus *Staphylococcus* (Figure 3) revealed that endophytes were distributed in six main clusters. Cluster 1 included sequences from grapevine leaf endophytes strictly related to the HAP species *S. saprophyticus* and *S. haemolyticus*, and to the non-pathogenic species *S. succinus, S. xylosus, S. lugdunensis*, and *S. warneri*. Cluster 2 included sequences from grapevine leaves and stems grouping with the pathogenic *S. aureus*, and one OTU from leaf strictly related to non-pathogenic species *S. sciuri*. The majority of OTUs from grapevine leaf and wood endophytic bacteria clustered together with the pathogenic species *S. epidermidis* (cluster 3). Cluster 4 included one OTU representing sequences from plant stems grouping closely to non-pathogenic species *S. caprae* and *S. urealyticus*. The remaining grapevine sequences, from both leaf and stem, grouped in two unassigned clusters (these OTUs shared sequence identity <97% in comparison with 16S rDNA gene nucleotide sequences previously deposited in GenBank). Blastn (best-hit) of these sequences indicated that the cluster 5 (represented by denovo411 and denovo2021) is related to *S. warneri*, while the cluster 6 (represented by denovo698, denovo2782, and denovo117) to *S. auricularis*.

Sequences identified as genus *Clostridium* (Figure 4) clustered into three groups relative to the tree inferred from the 16S rDNA sequence alignment. Most endophyte sequences in this genus were obtained from grapevine leaf. In detail, the first cluster included endophytic bacteria grouping with HAP such as *Clostridium difficile*, *C. tetani*, and with non-pathogenic environmental species (*C. vincentii* and *C. bijerinckii*). The second cluster included grapevine endophytic bacteria closely related to HAP *Clostridium botulinum* and *C. perfrigerans*, and non-pathogenic bacteria *C. drakei* and *C. ghonii*. The third cluster contained grapevine endophytic bacteria of the genus *Clostridium* related to unassigned species.

## Discussion

In the present study, we investigated microbial communities in the endosphere of grapevine leaves and stems by pyrosequencing and phylogenetic analyses of the 16S rDNA gene. Several endophytic OTUs belonging to four genera (*Propionibacterium, Staphylococcus, Clostridium*, and *Burkholderia*), known to include some species recognized as human- and animal-pathogens (HAP), were identified within the characterized microbial communities. Such OTUs were identified in enriched and not-enriched leaves and in grapevine stems. As reported in literature, the specific treatment of leaf samples with cell hydrolytic enzymes (microbe enrichment strategy) releases all microbes living in association with plant tissues (Jiao et al., 2006; Bulgari et al., 2009, 2011), improving the bacterial display and identification. The majority of the bacterial sequences in the genus *Propionibacterium*, from grapevine leaf and stem, were identified as *P. acnes.* This species is among the causing agents of acnes and its members are generally associated with human skin, where they feed on fatty acids secreted by sebaceous glands (Webster et al., 1981; Zouboulis, 2004). It also colonizes the human gut (Perry and Lambert, 2011), and is reported as an opportunistic pathogen in post-surgical infections (Nisbet et al., 2007). We previously identified genomic changes in *P. acnes* type Zappae, tightly associated with grapevines (Campisano et al., 2014). The wide diversity in sequences assigned to genus *Propionibacterium* shows that these endophytes are mostly present in the stem woody tissues (where the majority of sequences is amplified) and only marginally present in leaves, reinforcing the notion of a tight symbiosis of these bacteria with the plant.

The genus *Burkholderia* includes more than 60 species, some of which are known plant-dwellers (endophytes and epiphytes, Compant et al., 2008). Some species of *Burkholderia*, such as *B. mallei* and *B. cepacia* (*Burkholderia cepacia* complex, BCC), are recognized as HAP and plant pathogens (Govan et al., 1996; LiPuma, 1998; Coenye and Vandamme, 2003). Recently, *Burkholderia* species had gained considerable importance owing to their pathogenicity, but two findings had a strong impact on their ecological perception: (i) the identification of nitrogen fixation in *Burkholderia* species other than *B. vietnamiensis* (which belongs to the BCC), such as *B. brasilensis* M130 and *B. kururiensis*, and (ii) the description of legume-nodulating *Burkholderia* and their subsequent characterization as genuine endosymbionts (Suarez-Moreno et al., 2012). We found sequences clustering with such pathogenic *Burkholderia* in grapevine leaves. Stem-associated *Burkholderia* OTUs either grouped with non-pathogenic (or plant beneficial) species such as *B. phytofirmans* (Sessitsch et al., 2005) and *B. xenovorans* (Caballero-Mellado et al., 2007), or formed a separate phylogenetic group very similar to *B. kururiensis* and *B. sacchari*, by blastn analysis of best hit.

We can speculate that the presence of potentially pathogenic *Burkholderia* associated sequences in leaf green tissues but not in the woody stems represents a difference in the ecology of the plant-beneficial burkholderias from the HAP ones. All tissues in the leaf are in close proximity of the plant surface, while stem-associated tissues are possibly more difficult to colonize by transient colonizers. This may explain why plant-beneficial burkholderias were found mainly in the plant woody stem, while HAP ones only survive in leaves. We also noted that highly represented *Burkholderia* OTUs belong to the cluster including plant-associated species *B. phytofirmans* and *B. xenovorans* or to the clusters including *B. kururiensis* and *B. sacchari*, while the potentially pathogenic ones are in much lower numbers. *B. phytofirmans* is one of most studied endophytes. This strain was visualized colonizing the grapevine root surface, entering the endorhiza and spreading to grape inflorescence stalks, pedicels and then to immature berries through xylem vessels (Compant et al., 2008). We also reported in this work as one of the most abundant burkholderias in grapevine.

OTUs from grapevine leaves were mostly identified as genera *Clostridium* and *Staphylococcus*. These taxa include endophytic OTUs closely related to the pathogenic species *Staphylococcus saprophyticus, S. aureus*, and *S. epidermidis*. Quite interestingly, most *Staphylococcus* sequences appeared to associate taxonomically with *S. epidermidis* (Figure 3). *S. epidermidis* colonizes the epithelial surfaces of every human being. Furthermore, it is one of the most common causes of nosocomial infections. In addition to the abundant prevalence of *S. epidermidis* on the human skin, this high incidence is mainly due to the exceptional capacity of *S. epidermidis* to stick to the surfaces of indwelling medical devices during device insertion (Otto, 2008, 2009). At our knowledge this is the first report of pathogenic *Staphylococcus* associated sequences in plants.

Almost all the endophytic sequences assigned to the genus *Clostridium* were amplified from grapevine leaf DNA. This group included sequences grouping with HAP such as *Clostridium difficile*, *C. perfrigens, C. botulinum*, and *C. tetani*. A major factor important to the colonization of plants is how long bacteria persist in the soil before dying. *Clostridium* spores persisted in soil for 16 months and were found on the leaves of parlsey grown in the contaminated soil (Girardin et al., 2005). We can speculate that the presence of *Clostridium*, human and animal pathogenic taxa (HAPT) in grapevine leaves could be related to long–lasting spore contamination. Honey sometimes contains spores of *C. botulinum*, which may cause infant botulism in humans 1 year old and younger. The toxin eventually paralyzes the infant's breathing muscles (Tanzi and Gabay, 2002). *C. difficile* can flourish when other bacteria in the gut are killed during antibiotic therapy, leading to pseudomembranous colitis (a cause of antibiotic-associated diarrhea).

HAPT were indentified both in leaves and wood of grapevines. This depicts the ability of these pathogens to be internalized within plant tissues. Isolation of human pathogenic enterobacteria from within the tissue of fresh and minimally prepared produce has been reported (Eblen et al., 2004; Shi et al., 2007; Soto et al., 2007; Holden et al., 2009). Furthermore, it appears that once a plant has been colonized by bacteria there is the potential for vertical transmission to successive generations, as demonstrated for *S. typhimurium* on tomatoes (Guo et al., 2001). The exploration of endophytic communities, using metagenome-based community analyses (Bulgarelli et al., 2012; Lundberg et al., 2012) coupled with the exploration of the pathogenic potential of pathogens, are beginning to reveal that many HAP are capable of exploiting plant hosts. This means the apparent pathogens may have adapted to the plants and have become plant symbionts, for at least one stage of their life cycle. This also shows the ability and potential of HAP to persist on multiple hosts, with plants serving as intermediate hosts or reservoirs for them. The ability of these pathogens to maintain their population levels in a variety of environments likely increases their pan genome and evolutionary potential (Campisano et al., 2014). Although we focused on four genera (*Propionibacterium, Staphylococcus, Clostridium*, and *Burkholderia*) only, we identified several other taxa known for harboring HAP bacteria. Our analysis highlighted the presence of potential HAPT in the grapevine endosphere and, to the authors' knowledge, represents the first report of the unexpected occurrence of these bacterial taxa in this atypical (yet crucially important for agriculture) environment.

### Conflict of interest statement

The authors declare that the research was conducted in the absence of any commercial or financial relationships that could be construed as a potential conflict of interest.

## Footnotes

^1^http://www.liv.ac.uk/media/livacuk/centreforgenomicresearch/The_GS_FLX_Titanium_Chemistry_Extended_MID_Set.pdf
